# Development of village doctors in China: financial compensation and health system support

**DOI:** 10.1186/s12939-016-0505-7

**Published:** 2017-07-01

**Authors:** Dan Hu, Weiming Zhu, Yaqun Fu, Minmin Zhang, Yang Zhao, Kara Hanson, Melisa Martinez-Alvarez, Xiaoyun Liu

**Affiliations:** 1 China Center for Health Development Studies, Peking University, Beijing, China; 2 London School of Hygiene and Tropical Medicine, London, England

**Keywords:** Barefoot doctor, Community health worker, Compensation, Health system, Village doctor

## Abstract

**Background:**

Since 1968, China has trained about 1.5 million barefoot doctors in a few years’ time to provide basic health services to 0.8 billion rural population. China’s Ministry of Health stopped using the term of barefoot doctor in 1985, and changed policy to develop village doctors. Since then, village doctors have kept on playing an irreplaceable role in China’s rural health, even though the number of village doctors has fluctuated over the years and they face serious challenges. United Nations declared Sustainable Development Goals in 2015 to achieve universal health coverage by 2030. Under this context, development of Community Health workers (CHWs) has become an emerging policy priority in many resource-poor developing countries. China’s experiences and lessons learnt in developing and maintaining village doctors may be useful for these developing countries.

**Methods:**

This paper aims to synthesis lessons learnt from the Chinese CHW experiences. It summarizes China’s experiences in exploring and using strategic partnership between the community and the formal health system to develop CHWs in the two stages, the barefoot doctor stage (1968 –1985) and the village doctor stage (1985-now). Chinese and English literature were searched from PubMed, CNKI and Wanfang. The information extracted from the selected articles were synthesized according to the four partnership strategies for communities and health system to support CHW development, namely 1) joint ownership and design of CHW programmes; 2) collaborative supervision and constructive feedback; 3) a balanced package of incentives, both financial and non-financial; and 4) a practical monitoring system incorporating data from the health system and community.

**Results:**

The study found that the townships and villages provided an institutional basis for barefoot doctor policy, while the formal health system, including urban hospitals, county health schools, township health centers, and mobile medical teams provided training to the barefoot doctors. But After 1985, the formal health system played a more dominant role in the CHW system including both selection and training of village doctors.

China applied various mechanisms to compensate village doctors in different stages. During 1960s and 1970s, the main income source of barefoot doctors was from their villages’ collective economy. After 1985 when the rural collective economy collapsed and barefoot doctors were transformed to village doctors, they depended on user fees, especially from drug sale revenues. In the new century, especially after the new round of health system reform in 2009, government subsidy has become an increasing source of village doctors’ income.

**Conclusion:**

The barefoot doctor policy has played a significant role in providing basic human resources for health and basic health services to rural populations when rural area had great shortages of health resources. The key experiences for this great achievement are the intersection between the community and the formal health system, and sustained and stable financial compensation to the community health workers.

## Background

The Sustainable Development Goals (SDGs) signed by governments at the United Nation General Assembly in September 2015 have committed ‘to ensure healthy lives and promote well-being for all at all ages’ by 2030. To achieve these health related goals, the SDGs suggested to ‘increase the recruitment, development, training and retention of the health workforce in developing countries’. Within this context, community health workers (CHWs) are experiencing a resurgence of interests to achieve Universal Health Coverage (UHC) [1].

CHWs have been a key component of health care delivery in many countries in the world. The World Health Organization (WHO) defined CHWs as members who live in the communities, are selected by the communities, are answerable to the communities for their activities, are supported by the health system but are not necessarily a part of its organization, and have shorter training than professional workers [2]. CHWs are widely used in settings with poor resources, where it would be impossible to train highly qualified health professionals in a short period of time [3]. They typically perform one or more functions associated with health care delivery, even though they usually have no formal professional certification. Evidence shows that CHWs have had an important role in increasing essential services’ accessibility to improve childhood survival and address other health priorities in specific settings [4].

China’s rural health system has always had a primary health care (PHC) focus. In 1960–70s, China established its rural primary health care system, including the “barefoot doctors,” the (old) cooperative medical scheme, and the three-tiered service delivery system at county, township, and village levels. Barefoot doctors were farmers who received minimal basic medical and paramedical training and worked in rural villages in China. Their purpose was to bring health care to rural residents. In the early 1980s, China stopped using the term “barefoot doctor” and replaced it with “village doctor.” By the end of 2013, China had 1.08 million village doctors. For the past 60 years, barefoot doctors and village doctors have played an important role in providing essential and preventive health care to rural population [5].

There is emerging recognition that CHWs function at the intersection of two dynamic and overlapping systems – the formal health system and the community [6]. A strategic partnership between communities and health systems must be built for CHW programmes to achieve their designed goals.

This study aims to synthesis lessons learnt from the Chinese CHW experience. It summarizes China’s experiences in exploring and using strategic partnership between the community and the formal health system to develop CHWs in the two stages: the barefoot doctor stage (1968 –1985) and the village doctor stage (1985-present). This analysis may have policy implications not only for its current health system reform, but also for other low and middle income countries who have difficulties in staffing their PHC services to achieve UHC.

## Methods

The main method used in this study is literature review. The study subjects of this review are health workers from village clinics, originally called “barefoot doctors,” later changed to “village doctors.” The review was based on published journal articles, policy documents, and books. Various types of studies, including qualitative studies, cross sectional studies, reviews, and expert opinions/commentary/narrative paper were included in the analysis.

### Literature search

PubMed, Proquest, Google Scholar (first 50 pages) were used to search English papers. Additionally, two datasets (CNKI and Wanfang) were used to search Chinese papers. For policy documents, website and archived policies documents of Ministry of Health (MoH), Ministry of Education and other ministry or provincial administration were searched. Published and unpublished research reports and books were obtained based on experts’ suggestions.

Three types of terms were applied in data search of both English and Chinese literature: 1) terms about health workforce: barefoot doctors, village doctors, community health workers, lay health workers, mid-level health workers, and allied health professionals; 2) Work settings terms: primary health care, village clinic, township health centers, community health centers, community health stations; 3) Territory terms: China.

**Fig. 1:**
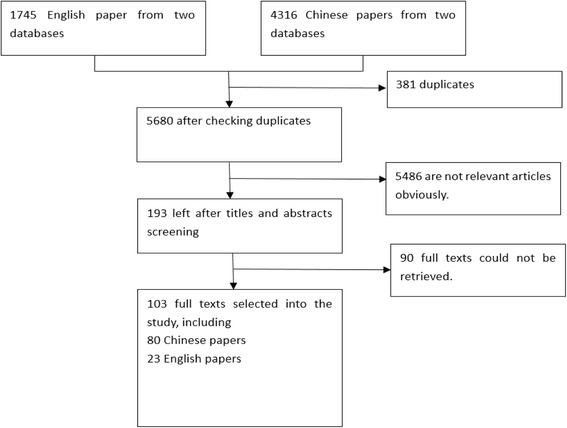
Literature search and selection process

In total, 103 papers were selected in the review, including 80 papers published in Chinese journals and 23 in English journals (Fig. 1). In addition, 5 books about barefoot doctors and village doctors were also reviewed (4 in Chinese and 1 in English).

We used the mixed-method appraisal tool to assess the quality of selected papers [7]. Each paper was rated from the lowest one point to the highest four points as the indication of the paper’s quality. Among the 80 Chinese papers, quality of 18 papers were rated as two points, 33 papers were rate as three points, and 29 papers were rated as four points, All the 23 English papers and the five books were rated as four points. We included all relevant studies no matter the level of quality.

### Data extraction and synthesis

An analytical framework regarding the partnership strategies for communities and health system to support CHW development was used to guide data extraction and synthesis. The framework included four themes: 1) joint ownership and design of CHW programmes; 2) collaborative supervision and constructive feedback; 3) a balanced package of financial and non-financial incentives; and 4) a practical monitoring system incorporating data from the health system and community [6]. A data extraction tool was developed based on the analytical framework to facilitate the process of retrieving relevant information. Two reviewers extracted information from the selected literatures independently. The information extracted from the selected articles was then synthesized following the themes in the analytical framework. When possible, comparisons were made between barefoot doctor stage (1968–1985) and the village doctor stage (1985 to present).

Though all the selected 103 papers provided useful information for the study, the review mainly refers to those papers which are closely related to the four themes in the framework.

## Results

The results starts with a brief introduction of policy process that led to the introduction and the continuing implementation of the programme on barefoot doctors. It then describes the strategic partnership between the community (village) and the health system in developing the CHWs, with special focus on financial compensation.

### Development history of barefoot doctors and village doctors

China’s CHW development included two stages, barefoot doctor stage (1968 to 1985) and village doctor stage (1985 to present). The following section will briefly introduce the policy implementation process, the policy contents, and some policy context.

#### Barefoot doctor stage (1968 to 1985)

China started to train rural health workers with different titles (health aides, maternal and child health aides, and nursing aides) in 1951 to address the great challenges of widespread infectious diseases and a serious shortage of medical services [8]. This policy to train lay health workers laid the foundation for the later policy to train barefoot doctors. In this study, these different types of grassroots rural health workers are all considered as community health workers.

In the mid-1960s, despite the economic growth, the disparity between urban and rural areas in China became a serious concern [9]. On June 26, 1965, Chairman Mao Zedong in his instruction on rural health work criticized the bias of health resource allocation towards urban cities and urged expansion of efforts to emphasize rural health [9]. He instructed that urban health professionals should coordinate outreach service to rural areas and help train rural health workers [9]. Consequently, some urban hospitals sent their mobile medical teams to rural areas to provide health services.

In 1968, the journal Red Flag published a paper on barefoot doctor program, which was widely believed as the starting point of a national policy to train paramedics quickly to meet rural health needs [10]. Since then, the barefoot doctor system had been expanding and received increasing attention. In 1976, the MoH held the first national conference on barefoot doctors to summarize experiences in training barefoot doctors [11]. In 1979, five ministries, including the MoH, jointly issued a document entitled Charters of the Rural Cooperative Medical Scheme, in which a special chapter clarified specific requirements for barefoot doctors. It regulated that there should be at least 2 barefoot doctors for each village, one of whom should be female [12]. In 1981, the State Council issued a policy document regarding the compensation of barefoot doctors. It stipulated that barefoot doctors, as intellectuals in rural areas, should be paid at the same level as rural teachers [13].

Barefoot doctors were recruited from the local rural villages. Young farmers with certain educational backgrounds were selected from poor and middle income classes to receive a 3–6 months medical training at commune or county levels. They came back to their home village after the short training to provide basic medical services, as well as public health services, to the rural population in their local villages. The barefoot doctor received a modest payment from the local collective economy [5].

#### Village doctors (1985-present)

In early 1980s when China started open policy and economic reform, the barefoot doctors could no longer meet the increasing demand of health services among rural population. In January 1985, the title of barefoot doctor was canceled by the MoH. From then on, a new title “village doctor” replaced it in the Chinese health system glossary [14, 15]. Barefoot doctors needed to pass an examination to be issued a village doctor certificate.

During the 1980s, along with the economic reform in China, the health system met new challenges in rural areas. The newly transformed village doctors were in a very unstable stage. They lost their financial support from the rural villages. Some village doctors left their jobs and went to urban areas to engage in commercial activities and other non-health related activities to make their living. The remaining village doctors had to financially heavily rely on revenues from drug sales on a fee for services basis and they stopped providing public health services as expected [11, 16].

Despite of these financial barriers, a new opportunity for the development of village doctors appeared in early 1990s when Chinese government started PHC initiatives to achieve its commitments on “health for all strategy”, village doctors had been playing important roles in the PHC initiatives. 10 out of the 13 indicators outlined in the PHC plan were directly or indirectly related village doctors (for example, indicators on coverage of safe water, and hygienic toilets). These PHC initiatives provided extensive training to village doctors and built up their capabilities [11]. In 1991, the State Council directed, in a rural health reform policy, that newly recruited village doctors should have at least three years’ medical education in the future [17]. However, the regulation was not properly implemented and unqualified village doctors continued to enter the team [11]. In 1997, the Central Committee of the Communist Party of China (CCCPC) and State Council stated in health reform and development policies that, the income level of village doctors should be no less than that of village officers [16]. However, this was not properly implemented, partly due to financial constraints of local governments. Income levels of rural teachers and village officers have continued to increase during the last 20 years, yet the compensation for village doctors has remained low. In 2003, the State Council issued its first and only law pertaining to village doctors to formalize its agenda to encourage greater development of village doctors in rural China [18]. The overall policy process of CHW is shown in Table 1.

**Table 1:** Development history of CHWs in rural China

Year	Events of CHW development in rural China
1951	China started in 1951 to train rural health workers with different titles (health aides, maternal and child health aides, and nursing aides).
1965	Chairman Mao urged more health resources should be allocated towards rural areas.
1968	The barefoot doctor program was introduced by the journal Red Flag as a national policy.
1976	First national conference on barefoot doctors for different provinces to share their experiences.
1979	MoH and other 4 ministries jointly issued Charters of the Rural Cooperative Medical Scheme, with specific requirement for barefoot doctors.
1981	State Council policy on compensation of barefoot doctors.
1985	MoH replaced the title of barefoot doctor with “village doctor”.
1991	State Council policy on PHC, village doctors played significant role.
1997	CCCPC and State Council health reform plan regulated the income level of village doctors should be no less than that of village officers.
2003	State Council issued its first and only law pertaining to village doctors to formalize its agenda to encourage greater development of village doctors in rural China.

### Strategic partnership between community and health system

The design and implementation of barefoot doctor and village doctor system in rural China proved to be a good example of collaboration between the community and the formal health system in developing CHWs.

#### Joint ownership and design of CHW programmes

In the design of barefoot doctor system, there was close collaboration between the health sector and the community. At national level, Chairman Mao was the champion to introduce and promote the barefoot doctor system. He believed that a paramedic cadre receiving a short period of training was a key policy to solve the health and health care problems in rural areas. He had great political power to push policy development and implementation. Under his instruction, the MoH served as the implementing agency of barefoot doctor policy. Most of the policy documents were drafted, issued, and implemented by the MoH. Moreover, the Cooperative Medical Scheme CMS was implemented at township and village level, providing an institutional basis for barefoot doctor policy. The townships and villages leaders and members selected the barefoot doctors for training, and the formal health system, including urban hospitals, county health schools, township health centers, and mobile medical teams provided training to the barefoot doctors.

After 1985 when the village doctors started to replace the barefoot doctors, the formal health system played a more dominant role in the CHW system including both selection and training of village doctors, while the rural village gradually lost its role in designing and implementing the village doctor policy.

#### Collaborative supervision and constructive feedback

Supervision of barefoot doctors came from two sources. Technical supervision was provided by physicians from township health centers or more advanced and experienced barefoot doctors, while administratively, barefoot doctors were supervised by village and township officers [19]. In theory, barefoot doctors were elected by the village members, but in fact, they were usually appointed by the villages’ head, to whom they were accountable [20].

In the village doctor stage, supervision mainly come from the township health centers and county health bureaus. Some recent studies on the supervision of village doctors reported that supervision of village doctors was weak and ineffective [16]. Village doctors complained that the outcome of supervision by township staff sometimes influenced the amount of subsidy they received, and township health center directors mentioned that supervision was difficult to conduct [11].

#### A balanced package of financial and non-financial incentives

Over the past decades, one of the important characteristics is that there were various compensation mechanisms for village doctors at different stages [21].

### Compensation from the collective economy

During 1960s and 1970s, the main income source of barefoot doctors was from the collective economy of their villages. A “work point” system was applied for the allocation of agriculture work and products. Barefoot doctors could earn certain amount of work points from provision of health services. Similar to the farmers, they could also have work points for their agriculture labor work when not providing health services. Generally, the daily work points of health service from barefooted doctors were equivalent to village officers and teachers, which were higher than the average level of other rural residents [15]. Barefoot doctors were compensated with a modest income, which the collective economy of the village could afford.

### Compensation from charge of health services

After the transition from barefoot doctors to village doctors, how to provide compensation for village doctors became a heated debate [16, 22]. During the 1980s, village doctors lost their financial support from the community, and they began to heavily depend on charges of health services, especially from sale revenues of drugs. Their income had significant variations among different places. A study in 2006 showed that the average annual income of a village doctors was CNY 4629, (about USD 730) in a rural county in Chongqing and CNY 20,000, (about USD 3200) in Pudong District of Shanghai [11]. With financial incentives from the service charges, village doctors were motivated to deliver clinical services for revenues other than being passionate to deliver public health services.

### Compensation from government subsidies

Since the 21st century, especially after the new round of health system reform in 2009, government subsidy has become an increasing source of village doctors’ income. This includes subsidies for essential medicines and essential public health services package. For example, the central government subsidized CNY 5 per capita to compensate village doctors to implement the essential medicine policy. In Sichuan Province, a village doctor can obtain CNY 6375, (about USD 1000) annually from government subsidies accounting for about 17.1% of total annual income, and the rest will be gained from service charges [22].

Given that barefoot doctors only had a modest income, they were often motivated by nonfinancial incentives. First, they enjoyed trust and respect from the local rural residents for various cultural reasons [9]. Second, barefoot doctors also gained good reputations through propaganda. There was an abundance of poems, novels, and films introducing and praising specific barefoot doctors and the whole system. Some model barefoot doctors were well known throughout the country [9].

In terms of career development, there was no clear career pathway for barefoot doctors and village doctors. They could not be covered by the existing health professionals’ career pathways due to their special subset of knowledge/skills and their multiple tasks. Therefore, special professional titles for this group of CHWs were not designated [10].

#### A practical monitoring system incorporating data from the health system and community

The workload of barefoot doctors was calculated as work points, and the data were mainly from the village. There was no report found regarding the monitoring system of barefoot doctors’ services.

Monitoring of public services provision has been developed since 2009’s health reform. The essential package of public health services was one of the main reform components. Financing of essential public health services are capitation based. Village doctors are main providers of essential public health services. A performance based payment system was implemented in order to allocate the budget to village doctors. The quantity and quality of public service provision of village doctors were closely monitored within the system on a monthly basis [23].

## Discussion

While collaborations between the community and the formal health system in developing CHW system receive increasing attentions in international literature [6], we did not find any analysis from this perspective in China’s CHW development. This review found that China’s history in developing barefoot doctors and village doctors may provide valuable experiences and lessons in this regard. The strategic partnership between the two sides seems to be more systematic in the barefoot doctor stage than in the village doctor stage. In the former stage, villages and township health centers worked more closely in designing the CHW system. There were dual supervision mechanism from both village and township health centers. Financial and nonfinancial incentives were well built into the community and the health system. However, in the latter stage, it seems that the formal health system has taken a more dominant role in implementing the CHW programme, while the communities step back and provide less support to the programme. In all the four areas, namely ownership and design, supervision, incentive, and monitoring, almost all policies and interventions are from the formal health system. The tie between village doctors and the local community are less tight than before. This may be one of the key reasons the village doctor system face various challenges in the health system [11].

One of the major mechanisms for the development of CHWs is sustained and stable financial compensation. When the government and local community cannot fully afford the cost of village doctors, it is feasible to allow them to charge user fees so that village doctors can be motivated and retained in the health workforce. It is possible that village doctors may over provide medical services or neglect public health services with this financial incentive. Therefore regulation and supervision of village doctors’ service provision behavior should be strengthened when user fees are introduced.

We also need to note the experiences and lessons of barefoot doctors and village doctors occurred in specific context. The barefoot doctor system was initiated, developed and eventually collapsed within a special political, economic and social context. The barefoot doctor system was organized within a collective economy context which could afford barefoot doctors’ modest salaries. When the collective economy lost its financial base and collapsed during the economic reform in early 1980s, barefoot doctors no longer received regular a salary from the collective economy, and became private practitioners, being responsible for profits and losses of their clinics [24]. Barefoot doctors were established in a specific social and cultural context where they enjoyed trust and respect from their local villagers [9, 25].

Although China has made great achievement of developing a large workforce of village doctors, the policy is also facing great challenges. The first challenge is the barriers for village doctors’ identity recognition in the health system. Since 1980s with the collapse of rural collective economy, village doctors have been gradually isolated from the rural community regarding their management and compensation. Although they are getting closer to the formal health system, they are never considered as a formal part of the system. For example, village doctors are not counted as health professionals in China’s health statistics. The second challenge is their low income. The central government has issued a few policies to guarantee village doctors’ compensation, but none has been well implemented due to unspecified funding sources and channels. The majority of village doctors do not have pension. The third challenge is the limitations in their qualification and career development. Due to their low educational level, most village doctors cannot pass examination to obtain a practice license of assistant physician. They have limited and unclear opportunities in career development. The in-service training they receive cannot effectively improve their knowledge and skill. Due to these above challenges, it is difficult to attract new qualified village doctors to work in rural areas.

## Conclusion

The village doctors have played a significant role in providing basic health workforce and basic health services to rural population when rural areas had great shortages of health resources. Sustained and stable financial compensation is one of the most important experiences for this great achievement. China applied various mechanisms to compensate village doctors at different stages, from the collective economy, charge for medical services, and government subsidies. Furthermore, as the grass-root health professionals, village doctors can only play its crucial role and function when closely integrated with rural community and the formal health system.

## Abbreviations

CCCPC: Central committee of the communist party of china
CHW: Community health worker
MoH: Ministry of health
PHC: Primary health care
SDG: Sustainable development goals
UHC: Universal health coverage
WHO: World health organization

## References

[1] Pettigrew LM, De Maeseneer J, Anderson M-IP, Essuman A, Kidd MR, Haines A (2015). Primary health care and the sustainable development goals. Lancet.

[2] World Health Organization (1987). Community health workers: pillars for health for all (report of the interregional conference, Yaoundé, Cameroon, 1-5 december 1986.

[3] World Health Organization (1989). Strengthening the performance of community health workers in primary health care: report of a WHO Study Group.

[4] Haines A, Sanders D, Lehmann U, Rowe AK, Lawn JE, Jan S (2007). Achieving child survival goals: potential contribution of community health workers. Lancet.

[5] Zhang D, Unschuld PU (2008). China’s barefoot doctor: past, present, and future. Lancet.

[6] Naimoli JF, Perry HB, Townsend JW, Frymus DE, McCaffery JA (2015). Strategic partnering to improve community health worker programming and performance: features of a community-health system integrated approach. Hum resour health.

[7] Pluye P, Robert E, Cargo M, Bartlett G, O’Cathain A, Griffiths F (2011). Proposal: a mixed methods appraisal tool for systematic mixed studies reviews.

[8] Ren R (2011). Development and function of village doctors in China. J Chinese Rural Health Serv Admin.

[9] Wen Y (2005). The emergence of the barefoot doctors and its social cultural backgrounds. J Yunan Nat Univ.

[10] Zhang R, Zhang W (2009). The rise and demise of the Chinese barefoot doctor. Chinese J Med Hist.

[11] Li F (2008). The history and current situation of villages doctors in China.

[12] MoH. Charters of the Rural Cooperative Medical Scheme. Beijing: Ministry of Health; 1979.

[13] MoH (1981). Report on reasonably addressing compensation issues of barefoot doctors.

[14] People’s Daily (1985). Stopping using the term barefoot doctors, Develop village doctors team.

[15] Li Y (2013). Thrive and collapse of barefoot doctors. Cult hist Wuhan.

[16] Tian J, Zhang G, Ren R (2010). Analysis on improving the income level and social security of village doctors under the background of new healthcare system reform. Chinese Health policy res.

[17] State council (1991). Instructions on reforming and strengthening rural health work.

[18] State council (2003). Regulation on the admiration of village doctors.

[19] Koplan JP, Hinman AR, Parker RL, Gong YL, Yang MD (1985). The barefoot doctor: Shanghai County revisited. Am J Public Health.

[20] Gong Y, Chao L (1982). The role of barefoot doctors. Am J Public Health.

[21] Chen Z, Wang Y, Cui X, Sun M, Li C, Wang H (2009). The origin, development and status quo of rural doctors in China. Chinese Prim Health Care.

[22] Gong Y, Yan F, Feng L (1997). Study on village doctors’ distribution, training and salary. J Chinese Rural Health Serv Admin.

[23] Zheng S (2013). Survey and analysis on current situation of rural doctors in Sichuan province. J Chinese Prim Health Care.

[24] Fang X (2012). Barefoot doctors and Western medicine in China.

[25] Yang N (2006). Re-making patients - Spatial politics under the conflict between Chinese and Western medicine (1832-1985).

